# Dynamic transmission model of routine mumps vaccination in Japan

**DOI:** 10.1017/S0950268818003230

**Published:** 2018-12-03

**Authors:** Taito Kitano

**Affiliations:** 1 Department of Neonatal Intensive Care Unit, Nara Prefecture General Medical Center, Nara, Japan; 2 Master of Public Health Degree Program, Johns Hopkins Bloomberg School of Public Health, Baltimore, Maryland, USA

**Keywords:** Cost-benefit analysis, immunisation programme, mumps vaccine, quality-adjusted life years

## Abstract

The mumps vaccine is not included in the national immunisation programme (NIP) of approximately 80 countries including Japan. To investigate the vaccine's cost-effectiveness, we developed a dynamic transmission model for routine one- and two-dose mumps vaccination programs in Japan. We calculated the incremental cost-effectiveness ratio compared with a current programme over a projected 50-year period. We created a Japanese population model and performed dynamic simulation to estimate the number of patients enrolled in the current programme, the routine one-dose programme, and the routine two-dose programme over the next 50 years using the Berkeley Madonna program. We estimated the medical and social costs of natural mumps infections and vaccinations to analyse cost-effectiveness. Finally, we performed a sensitivity analysis with parameters including vaccine cost, vaccine efficacy, medical costs per case, social costs per case, incidence of adverse events and discount rate. Base case analysis showed that both the one-dose and two-dose programmes predominated and that quality-adjusted life years (QALYs) were saved, compared with the current programme. The medical costs, total cost and QALYs saved during the study period in the two-dose programme compared with the current programme were 217 billion JPY, 860 billion JPY and 184 779, respectively. The two-dose programme surpassed the one-dose programme throughout the study period. In all the scenarios of the sensitivity analysis, two-dose vaccination was better than the one-dose programme. This simulation confirmed that the routine two-dose vaccination programme was more cost-effective and QALY-saving than either the one-dose programme or the current programme. Because of the variability of the results between the various models, further simulations with different models should be conducted.

## Introduction

In most countries, two-dose mumps vaccine programmes are an integral part of their national immunisation programmes (NIP). In these countries, the cost of the mumps vaccines is covered by governments. However, the mumps vaccine is not included in the NIPs of approximately 80 countries that have a huge burden of the disease, including Japan [1]. In Japan, the mumps vaccine has been excluded from the NIP because of vaccine-induced aseptic meningitis [2]. Over the past 25 years, Japan has not been able to restart the routine mumps vaccination programme even though the domestic vaccine strains have been improved and shown to cause aseptic meningitis with lower rate if the first dose vaccine is administered at 1-year old [3]. In the current programme, parents pay approximately 6000 JPY per each mumps vaccine for their children, which has led to low national coverage rate of the mumps vaccine (30–40% for the first dose) [4]. Because of that, the annual number of mumps infection in Japan is estimated to be from 400 000 to 1 500 000 with inconsequential number of mumps-induced complications such as meningitis, encephalitis, hearing loss, orchitis and oophoritis [5]. This mumps epidemic is causing significant burdens not only for patients but also for their families because most of mumps patients are children, and their parents need to take care of them. It leads to billions of medical and social costs each year in addition to loss of healthy lives [5].

The reasons the mumps vaccine is not included in the NIPs of these countries vary. One of the major concerns for vaccination programmes is cost-effectiveness. Though few studies using the dynamic transmission model have been implemented, the mumps vaccine has been shown in previous studies in Japan to be highly cost-effective by using static models and the Markov model [5, 6].

To investigate cost-effectiveness with a more advanced model, we developed a dynamic transmission model for routine one- and two-dose vaccination programmes in Japan using sensitivity analysis and calculated the incremental cost-effectiveness ratio (ICER) compared with a current model over a projected 50-year period. In the current programme, the mumps vaccine is not included into the NIP, and parents bear the cost of any vaccines if they want their children to be vaccinated. Once the vaccine is included into the NIP, the vaccine cost is covered by the government, and the government encourages parents to have their children vaccinated. In one-dose routine programme, only one dose of the vaccination is included into the NIP, but parents need to bear the cost of second dose. In two-dose routine programme, parents do not have to pay any cost of the vaccination.

## Materials and methods

### Overview of the dynamic transmission model

The dynamic transmission model was developed using current Japanese epidemiologic data from 2000 to 2016 to perform the cost-effectiveness analysis of routine one- and two-dose mumps vaccination programmes compared with the current programme, from a societal perspective, over a projected 50-year period, using sensitivity analysis. The model comprised both epidemiologic and economic components. We used current basic epidemiologic and economic data from national surveillance and our own previous study [5, 7].

A literature review was conducted using the PubMed database and Igaku Chuo Zasshi database to select indicators for the model. It was programmed in Berkeley Madonna version 8.3.18 (Berkeley, CA, USA) and Microsoft Excel 2016 (Redmond, WA, USA). Berkeley Madonna software was used to estimate the number of mumps cases over a 50-year period using the current programme, routine one-dose programme and routine two-dose programme. A cost-effectiveness analysis with the ICER calculation was conducted using Excel 2016. All the simulations and calculations were performed using the methods described below.

### Epidemiologic component

**Fig. 1.**
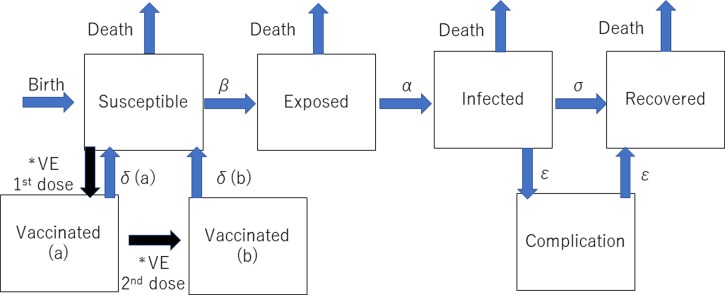
Structure of the dynamic transmission model. VE= vaccine efficacy, *β* = transmission rate, *α* = latency, *σ* = recovery rate, *δ*(*a*) = waning rate for one dose, *δ*(*b*) = waning rate for two doses, *ε* = complication incidence. *The event happens only once.

The dynamic transmission model was created after a thorough review of previous studies of the mumps vaccine with the aim of making the model as realistic as possible. We used the deterministic SEIR (Susceptible-Exposed-Infected-Recovered) model on Berkeley Madonna programming (Fig. 1). We created 13 age categories (0, 1, 2, 3, 4, 5, 6, 7, 8, 9, 10–14, 15–19 and >20 years) to simulate the real Japanese population. We inputted into the model the national population data for each age category [8, 9]. The annual number of births was assumed to be 1 000 000 at year 0, decreasing by 10 000 per year and the initial annual number of deaths was 13 000 000, projected to increase by 10 000 per year over the next 50 years to simulate the expected future Japanese population [5, 6]. In the model, the life expectancy of all individuals was 85 years. All were born susceptible until they became vaccinated or infected with mumps. The average infection period was assumed to be 1 week in all cases. Once a susceptible person became exposed, they moved to infected status after average 17 days of latency period. In the study, we did not consider asymptomatic patients because of the lack of immunity data of asymptomatic infection. Then, all the infected individuals moved to recovered status with permanent immunity after 1 week of infected status, with a small proportion of permanent sequelae (encephalitis and hearing loss). For the incidence of each mumps complication, the data from our previous study were used [5]. The details of each indicator used in the programme are shown in Table 1.

**Table 1.** Parameters of epidemiologic and economic components of the model

		Base case
Vaccine coverage (first/second dose)	Current programme	35%/5%
	Routine one-dose programme	95%/5%
	Routine two-dose programme	95%/90%
Vaccine efficacy immediately after immunisation	First dose	84%
	Second dose	90%
Waning rate	First dose	2% per age quintile before 4 years and 10% per age quintile after 4 years
	Second dose	2% per age quintile before 4 years and 10% per age quintile after 4 years
Average life expectancy		85 years
Average period of infection		7 days
Initial incidence of the programmes^a^		714.93/100 000 person-years
Average medical costs per infection		¥23 786
Average social costs per infection		¥50 278
Average QALYs lost per infection		0.0108
Vaccine costs		¥6000
Average medical costs per shot due to adverse event		¥170
Average medical costs per shot due to adverse event		¥543
Parental nursing costs for vaccination per shot		¥6699
Discount rate		3%

The methodology for the calculation of each indicator was described in our previous study [2].

a Initial incidence was the estimate of the average incidence from 2000 to 2016.

The incidence of infection (d*I/*d*t*) was estimated using the following formula, defined by the transmission rate (*β*), the size of the susceptible population (*S*), the size of the infected population (*I*) and the size of the entire population (*N*):

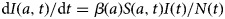


All susceptible individuals were assumed to have contact with persons of all age groups at the same rate, irrespective of their age. The transmission rate of each age quintile was estimated based on the age-specific national annual incidence divided by the age-specific susceptible population and the prevalence from 2000 to 2016 [7, 8]. The number of susceptible population was estimated by the number of total population subtracted by the number of vaccinated, exposed, infected and recovered population in each age group. Because Japan uses a sentinel reporting system instead of a census report, the estimates for the total and age-specific incidence from 2000 to 2016 were used as the initial incidences for the programmes of this study [7].

Two types of vaccinated statuses were created in the model. All the children who had first and second dose vaccinations were assumed to have received them at ages 1 and 5 years. If the children had been successfully immunised with the initial dose, they were assumed to have moved to the Vaccinated (a) stage in the model, and if the second dose successfully boosted their immune status, they moved to Vaccinated (b) stage. Based on a previous study showing that the vaccine efficacy of Japanese strains (Hoshino and Torii strains) was 78–90% for one-dose, the vaccine efficacy of one dose was estimated to be 84% (78–90% in the sensitivity analysis) immediately following immunisation in this study, but this waned over time [10]. This previous study involving Japanese strains showed that the efficacy was better than that of the Jeryl-Lynn strain [10–14]. Because there are no data regarding the two-dose vaccine efficacy of the Japanese strains, a wide-range sensitivity analysis of the second dose vaccine efficacy was performed. Vaccine efficacy is much higher in the first 4–5 years following vaccination [11–14]. Therefore, the rate of immunity loss was estimated to be 2% per year for the first 4 years after vaccination and 10% per transfer of one age quintile afterwards. Once the individuals lose immunity from vaccination and become infected, the severity of infection is assumed to be the same as that of non-vaccinated individuals in the model because there are no accurate data regarding the severity of mumps infection for vaccinated individuals.

Vaccine coverage rates for the current programme (no routine vaccination) were estimated at 35% and 5% for the first and second dose, respectively, throughout the study period. In contrast, first and second dose coverage rates were assumed to be 95% and 90%, respectively, in the routine vaccination programmes. In the routine one-dose vaccination programme, first and second dose coverage rates were assumed to be 95% and 5%, respectively, and the rates of the routine two-dose vaccination programme were 95% and 90%, respectively, consistent with the current measles-rubella vaccine coverage rate in Japan [15]. No catch-up programme was considered in our routine programmes. Therefore, the vaccine coverage rates at age 5 years for the first and second doses were 35% and 5%, respectively, for the first 4 years, and these became 95% and 5%, respectively, after year 4 in the routine one-dose programme. In the routine two-dose programme, the vaccination rates were 55% and 35%, respectively, for the first 4 years and 95% and 90%, respectively, after year 4.

The overall model is shown in Figure 1, and the details of the programme for Berkeley Madonna programming are shown in Appendix A. We used the data comprising the average number of mumps cases from 2000 to 2016 as the number in the infected population at the beginning of the simulation in addition to the incidence of each complication, and the incidence of the vaccine-induced adverse events from previous studies [5]. Other parameters of the epidemiologic component are shown in Table 1.

### Economic component

To create an accurate economic model, the following costs were considered: vaccine costs, the medical costs of natural infection, the medical costs of adverse events due to vaccination and the social costs from parental productivity loss for nursing due to children's natural infections, vaccine-induced adverse events and time used for taking children to be vaccinated (4 h/dose). The vaccine costs were estimated to be 6000 JPY per shot, including the costs for the healthcare providers. Because there is currently no approved measles-mumps-rubella vaccine in use in Japan, the scenario of independent vaccinations was considered the base case. Although productivity losses of parents were counted as social cost, productivity losses of patients younger than 20 years old were not counted in the cost calculation because these can be double-counted as part of the burden of disease. The quality-adjusted life years (QALYs) loss was assessed by each patient's quality of life lost through natural infection and adverse events due to vaccinations. QALYs loss and the medical and social costs per infection in addition to the vaccine-induced adverse events and average hourly wage were estimated using the same values as in the previous study [5]. The discount rate of the base case was 3% for both the economic and health indicators. The parameters of the model are shown in Table 1.

### Sensitivity analysis

We performed a sensitivity analysis with the following parameters: vaccine cost (4000–8000 JPY per dose), vaccine efficacy immediately following vaccination (78%/78% to 90%/96% for first/second doses), medical cost per case (+50 to −50%), social costs per case (+50 to −50%), incidence of adverse events (+50 to −50%) and the discount rate (0–6%). The ranges of these parameters were based on the previous study with the static model, the recent national epidemiological data and the previous literature about discount rate [5–8, 16–20].

## Results

### Model validation

**Fig. 2.**
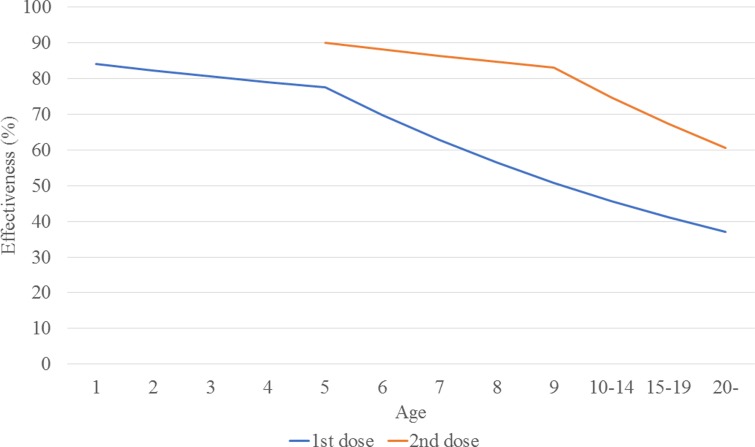
Estimated vaccine effectiveness, by age.

The calculated transmission rates (by age) in the base case scenario are shown in Table 2. The age-dependent vaccine effectiveness is shown in Figure 2. The range of base case scenario in the current programme was from 309 to 2289/100 000 person-years, whereas the real range of total incidence rate from 2000 to 2016 by national data was from 245 to 1780/100 000 person-years. The range of base case scenario was compatible with that of total incidence. Both of real incidence and base case scenario in the model had periodical epidemics every several years. These mean that base case scenario is an optimal simulation of real incidence over the next 50 years. The results of the base case scenario showed that the average estimated total mumps incidences over the first 5 years and the 50 years were 851 and 619 per 100 000 person-years, with a gradual decrease until year 50. The calculated average total incidence from 2000 to 2016 was 715 per 100 000 person-years. Considering the expected decrease in incidence in the future due to a population decrease in Japan, this model reasonably represented the estimated mumps epidemiology over the next 50 years.

**Table 2.** Mumps transmission rate in base case scenario, by age

Years	Transmission rate (/year)
0–1	133.1
1–2	830.3
2–3	1204.3
3–4	1721.0
4–5	2729.7
5–6	4091.1
6–7	4306.4
7–8	3873.5
8–9	3000.1
9–10	2026.7
10–15	458.7
15–20	23.6
20	1.1

Transmission rates by age were calculated based on the assumed base-case vaccine efficacy (84%/90%) and coverage rate (35%/5%).

d*I*(*a*, *t*)/d*t* = *β*(*a*) *S*(*a*, *t*)*I*(*t*)/*N*(*t*)

### Epidemiologic component

**Fig. 3.**
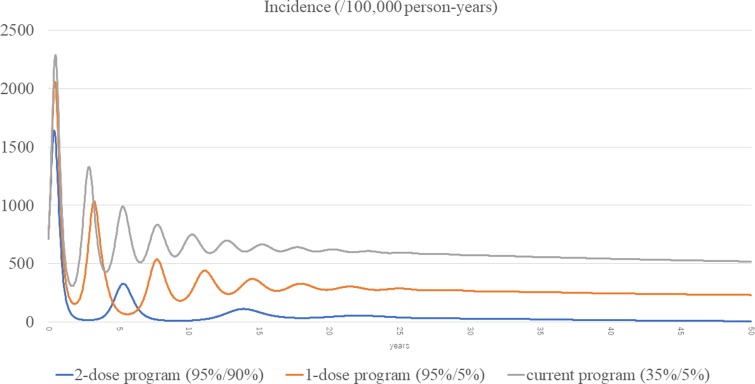
Incidences of the three scenarios.

The total incidences of the three scenarios (current, routine one-dose and routine two-dose programme) are shown in Figure 3. The estimated incidence in the current programme remained high, with periodic epidemics over the first decade. Although the incidence of the current programme decreased over time, it was still as high as 519 per 100 000 person-years at year 50. In the routine one-dose programme, the incidence showed some reduction with periodic epidemics; however, constant infections were seen throughout the study period, with a final incidence of 232 per 100 000 person-years at year 50. In the routine two-dose programme, the incidence declined dramatically immediately after the start of the programme. Although there was a small surge at year 5, it declined steadily with continuous suppression throughout the remainder of the period, and the incidence reached 7.22 per 100 000 person-years at year 50.

The age-specific incidences of the three scenarios are shown in Appendix B. The one- and two-dose scenarios showed overall declines in all the age groups. In the one-dose programme, the 6-years-old comprised the highest incidence population, whereas the 5-year-old were the highest incidence population in the current programme. In the two-dose programme, the incidences were very small in all the age groups after several years.

### Economic component

Considering that a small proportion of patients has complications, the average medical and social costs per one infection were 23 786 and 50 278 JPY, respectively [5]. The results of the cost-effectiveness analysis are shown in Table 3. In the base case scenario, the medical and total costs saved during the study period in the one-dose programme compared with the current programme were 154 billion and 555 billion JPY, respectively. The respective costs in the two-dose programme compared with the current programme were 217 billion and 860 billion JPY. The QALYs saved over the 50-year period were 105 605 and 184 779 in the one- and two-dose programmes, respectively. In terms of the ICER, the one- and two-dose programmes surpassed the current programme, and the two-dose programme surpassed the one-dose programme throughout the study period.

**Table 3.** The costs saved in the one- and two-dose programme compared with the current programme

	Case avoided	QALYs saved	Medical costs saved^a^	Total costs saved^a^	ICER
One-dose					
1 year	215 305	2277	1 239 218 493	7 526 581 950	Dominant
3 years	1 063 490	10 891	13 295 521 200	51 767 961 863	Dominant
5 years	1 449 907	14 498	14 929 968 265	62 987 315 974	Dominant
10 years	4 278 636	39 477	56 174 885 816	204 732 483 357	Dominant
30 years	11 740 164	85 486	123 750 767 388	447 846 351 820	Dominant
50 years	17 605 237	105 605	153 927 338 054	555 497 019 872	Dominant
Two-dose					
1 year	577 135	6118	4 346 214 362	22 397 270 764	Dominant
3 years	2 065 266	21 172	20 813 038 103	89 865 125 358	Dominant
5 years	3 496 965	34 982	35 962 571 537	151 851 348 403	Dominant
10 years	7 251 160	67 493	74 385 656 931	303 678 517 824	Dominant
30 years	20 528 319	149 217	172 909 591 401	689 481 315 593	Dominant
50 years	30 936 846	184 779	217 123 915 205	860 233 978 313	Dominant

a Unit; Japanese Yen.

### Sensitivity analysis

**Fig. 4.**
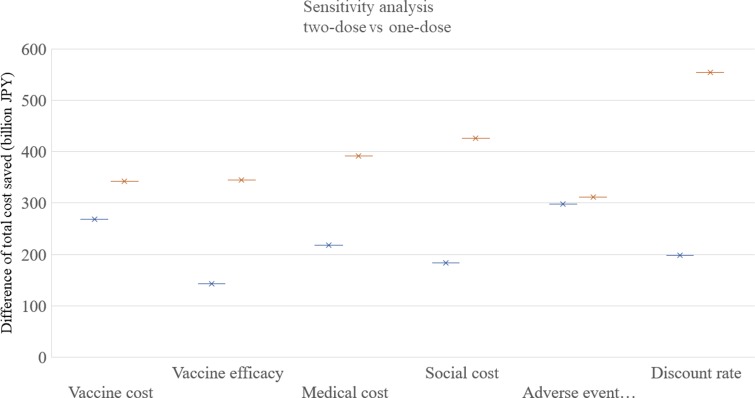
Sensitivity analysis. One-way sensitivity analyses were performed as following ranges of each factor: vaccine cost: 4000–8000 JPY, vaccine efficacy: 78%/78% to 90%/96% for first/second doses, medical cost: +50% to −50%, social cost: +50% to −50%, adverse event incidence: +50% to −50%, discount rate; 0–6%. JPY, Japanese Yen.

The results of the one-way sensitivity analysis are shown in Figure 4. Among the six parameters in the sensitivity analysis, the discount rate was the most sensitive, and the adverse event incidence was the least sensitive parameter. The routine two-dose programme dominated both the routine one-dose programme and the current programme in all the scenarios in the one-way analysis.

## Discussion

In this analysis, the dynamic transmission model was used to simulate the routine one- and two-dose mumps vaccination programmes in Japan. We found significant cost and QALYs savings in the routine two-dose programme. The dynamic transmission model is often used to simulate the future epidemiology of vaccine-preventable infectious diseases because it includes the consideration of herd immunity [21]. It is used in simulations for many types of vaccines [22–25]. In terms of mumps vaccination, most of the simulations previously reported were based on static models [5, 26, 27]. Because many developing countries have not yet added mumps vaccination to their NIPs, this study can be useful for the improvement of their NIPs in addition to previous data supporting the two-dose vaccines from developed countries where the two-dose mumps vaccines were already included into the NIP [28, 29]. Our study used a deterministic model, where all the variables were determined by the parameter values, whereas some other dynamic transmission studies have adopted a stochastic model [30]. The deterministic model is more suitable for large populations and is easier to calibrate than the stochastic model [21]. Although we tried to make our estimations as accurately as possible, no model can operate the same in the real world. Therefore, variations on the simulations may yield different results.

If the ICER is less than one national GDP per capita, the intervention is considered cost-effective under the WHO guidelines [31]. In our study, the ICERs of the routine two-dose programme surpassed the routine one-dose programme and the current programme throughout the study period. In many simulations of vaccine programmes, a slight increase in the burden of disease is seen after the introduction of vaccinations [22]; however, this analysis showed that the benefit of mumps vaccination is so substantial that there is a consistent benefit in the costs and QALYs from the routine vaccination programmes (including in year 1). Therefore, the cost-effectiveness of the routine two-dose vaccination is apparently very high. It was concerning that the age shift of infection due to vaccination may have caused a higher incidence of complications. In our analysis, the age-specific incidences of the routine two-dose programme were smaller across all age groups compared with those of the routine one-dose programme and the current programme. In some guidelines, a differential discounting between the health and economic indicators is recommended [16, 17]. However, we adopted equal discounting because Japan does not specifically recommend differential discounting. Because our results showed that the routine two-dose programme consistently dominated the other two programmes, discounting the health outcomes at a different rate from the economic ones would not have affected the final results. Some experts argue that non-constant discounting should be applied in the cost-effectiveness analysis, particularly in vaccine programme simulations [18, 19]. However, these methods of discounting remain controversial [20].

Our study had several limitations. First, the data regarding the efficacy of the mumps vaccine, particularly the Japanese strains, are lacking. For example, there are no data regarding the efficacy of the second vaccine dose or about the waning rate of immunity after vaccination against the Japanese strains. Although the vaccine efficacy had a relatively small effect on our results, the lack of data remains a major concern in making the simulation as accurate as possible. Second, our model did not reflect outbreaks after the population becomes highly immunised with the two-dose vaccination. Because there remain some mumps outbreaks in countries where two-dose coverage rates are high (>90%), small outbreaks would still occur even if most of the Japanese population has received the two-dose vaccination [32–34]. However, there are several factors that support the notion that Japan might not experience outbreaks as do most developed countries. Japanese strains have a better vaccine efficacy than that of Jeryl Lynn, though the data on the efficacy of the second dose is lacking. In addition, instead of the Jeryl Lynn strain, type A, the genetic type of the Japanese strains is type B, which is expected to have a more cross-reaction with the type G mumps virus, accounting for most outbreaks in the developed world [35]. Third, this model assumed that the entire susceptible population had the same contact rate across all age groups, but the assumption was unrealistic because each age group has a different contact rate. For example, young children and adolescents are more likely to become infected at their kindergartens or schools. Therefore, a different contact matrix would create a different result. Fourth, we estimated that the vaccine coverage in routine programmes achieved their targets immediately after the start of the programmes; however, in the real world, coverage might gradually increase and achieve the target rate several years after programme implementation.

Several factors could have affected our results. A consideration of catch-up programmes would have shown a more rapid decline in the incidence of disease. Simultaneous inoculation with other vaccines, such as the measles and rubella vaccine, would have decreased the social burden for parents to take their children to clinics, resulting in a more vaccine-favourable outcome. Estimating that the severity of each complication due to adverse events was less than that of natural infection would also have resulted in a more vaccine-favourable outcome. One may also consider asymptomatic mumps, which, as a previous study reported, is not uncommon, particularly in infants [36]. However, none of these factors stated above do not significantly affect our conclusion that the two-dose mumps vaccine programme is highly cost-effective.

In conclusion, our results showed that there are significant benefits of the routine two-dose mumps vaccination programme. This cost-effectiveness analysis could be used as a basis for policy decisions not only in Japan but in other countries where mumps vaccine has not yet been incorporated as part of the NIP. Of note, however, is that the value of the variables should be modified to be applied to each distinct situation and setting because each region's disease burden and economic indicator is different.

## Supplementary material

For supplementary material accompanying this paper visit https://doi.org/10.1017/S0950268818003230. click here to view supplementary material
