# Supplementary material for: Dynamic transmission model of routine mumps vaccination in Japan
Source: Epidemiol Infect. 2018 Dec 3;147:e60. doi: 10.1017/S0950268818003230 (PMC6518553; doi:10.1017/S0950268818003230)
Supplement: Supplementary file 1 [file S0950268818003230sup001.docx]

Appendix A. Programming of base case in Berkeley Madonna

S; susceptible

I; infected

R; recovered

Va; vaccinated (a)

Vb; vaccinated (b)

beta; transmission rate

delta; waning rate

sigma; infectious period

Grow; growing number

C1; coverage rate of 1^st^ dose at 1 year old

C2a; coverage rate of 1^st^ dose at 5 years old

C2b; coverage rate of 1^st^ dose at 5 years old

VE; vaccine effectiveness of 1^st^ dose at 1 year old

VEa; vaccine effectiveness of 1^st^ dose at 5 years old

VEb vaccine effectiveness of 2^nd^ dose at 5 years old

METHOD RK4

STARTTIME = 0

STOPTIME=50

DT = 0.02

t=time

Intotal=Itotal/Ntotal

Itotal= I0+I1+I2+I3+I4+I5+I6+I7+I8+I9+I10+I15+I20

Ntotal=N0+N1+N2+N3+N4+N5+N6+N7+N8+N9+N10+N15+N20

Stotal= S0+S1+S2+S3+S4+S5+S6+S7+S8+S9+S10+S15+S20

Etotal=E0+E1+E2+E3+E4+E5+E6+E7+E8+E9+E10+E15+E20

Vtotal=V1+V2+V3+V4+V5a+V5b+V6a+V6b+V7a+V7b+V8a+V8b+V9a+V9b+V10a+V10b+V15a+V15b+V20a+V20b

Rtotal= R0+R1+R2+R3+R4+R5+R6+R7+R8+R9+R10+R15+R20

d/dt(S0) = Births - S0 -beta0*S0*Itotal/Ntotal

d/dt(E0) = beta0*S0*Itotal/Ntotal -alfa*E0 -E0

d/dt(I0) = alfa*E0 -sigma*I0 -I0

d/dt(R0) = sigma*I0 - R0

d/dt(S1) = S0*(1-C1+C1*(1-VE)) - S1 -beta1*S1*Itotal/Ntotal + delta*V1

d/dt(E1) = beta1*S1*Itotal/Ntotal -alfa*E1 -E1 +E0

d/dt(I1) = alfa*E1 -sigma*I1 -I1 +I0

d/dt(R1) = sigma*I1 - R1 +R0

d/dt(V1) = -delta*V1 + S0*C1*VE -V1

d/dt(S2) = S1 - S2 -beta2*S2*Itotal/Ntotal + delta*V2

d/dt(E2) = beta2*S2*Itotal/Ntotal -alfa*E2 -E2 +E1

d/dt(I2) = alfa*E2 -sigma*I2 -I2 +I1

d/dt(R2) = sigma*I2 - R2 +R1

d/dt(V2) = -delta*V2 -V2+V1

d/dt(S3) = S2 - S3 -beta3*S3*Itotal/Ntotal + delta*V3

d/dt(E3) = beta3*S3*Itotal/Ntotal -alfa*E3 -E3 +E2

d/dt(I3) = alfa*E3 -sigma*I3 -I3 +I2

d/dt(R3) = sigma*I3 - R3 +R2

d/dt(V3) = -delta*V3 -V3 +V2

d/dt(S4) = S3 - S4 -beta4*S4*Itotal/Ntotal + delta*V4

d/dt(E4) = beta4*S4*Itotal/Ntotal -alfa*E4 -E4 +E3

d/dt(I4) = alfa*E4 -sigma*I4 -I4 +I3

d/dt(R4) = sigma*I4 - R4 +R3

d/dt(V4) = -delta*V4 -V4 +V3

d/dt(S5) = (S4+V4)*(1-C2b-C2a+C2a*(1- VEa)+C2b*(1-VEb)) - S5 -beta5*S5*Itotal/Ntotal + delta5a*V5a + delta5b*V5b

d/dt(E5) = beta5*S5*Itotal/Ntotal -alfa*E5 -E5 +E4

d/dt(I5) = alfa*E5 -sigma*I5 -I5 +I4

d/dt(R5) = sigma*I5 - R5 +R4

d/dt(V5a) = (S4+V4)*C2a* VEa -delta5a*V5a -V5a

d/dt(V5b) = (S4+V4)* C2b*VEb -delta5b*V5b -V5b

d/dt(S6) = S5 - S6-beta6*S6*Itotal/Ntotal + delta6a*V6a + delta6b*V6b

d/dt(E6) = beta6*S6*Itotal/Ntotal -alfa*E6 -E6 +E5

d/dt(I6) = alfa*E6 -sigma*I6 -I6 +I5

d/dt(R6) = sigma*I6 - R6 +R5

d/dt(V6a) = -delta6a*V6a -V6a +V5a

d/dt(V6b) = -delta6b*V6b -V6b +V5b

d/dt(S7) = S6 - S7 -beta7*S7*Itotal/Ntotal + delta7a*V7a + delta7b*V7b

d/dt(E7) = beta7*S7*Itotal/Ntotal -alfa*E7 -E7 +E6

d/dt(I7) = alfa*E7 -sigma*I7 -I7 +I6

d/dt(R7) = sigma*I7 - R7 +R6

d/dt(V7a) = -delta7a*V7a -V7a+V6a

d/dt(V7b) = -delta7b*V7b -V7b+V6b

d/dt(S8) = S7 - S8 -beta8*S8*Itotal/Ntotal + delta8a*V8a + delta8b*V8b

d/dt(E8) = beta8*S8*Itotal/Ntotal -alfa*E8 -E8 +E7

d/dt(I8) = alfa*E8 -sigma*I8 -I8 +I7

d/dt(R8) = sigma*I8 - R8 +R7

d/dt(V8a) = -delta8a*V8a -V8a+V7a

d/dt(V8b) = -delta8b*V8b -V8b+V7b

d/dt(S9) = S8 - S9 -beta9*S9*Itotal/Ntotal + delta9a*V9a + delta9b*V9b

d/dt(E9) = beta9*S9*Itotal/Ntotal -alfa*E9 -E9 +E8

d/dt(I9) = alfa*E9 -sigma*I9 -I9 +I8

d/dt(R9) = sigma*I9 - R9 +R8

d/dt(V9a) = -delta9a*V9a -V9a+V8a

d/dt(V9b) = -delta9b*V9b -V9b+V8b

d/dt(S10) = S9 - Grow10*S10/N10 -beta10*S10*Itotal/Ntotal + delta10a*V10a/5 + delta10b*V10b/5

d/dt(E10) = beta10*S10*Itotal/Ntotal -alfa*E10 -Grow10*E10/N10 +E9

d/dt(I10) = alfa*E10 -sigma*I10 - Grow10*I10/N10 +I9

d/dt(R10) = sigma*I10 - Grow10*R10/N10 +R9

d/dt(V10a) = -delta10a*V10a/5 -Grow10*V10a/N10 +V9a

d/dt(V10b) = -delta10b*V10b/5 -Grow10*V10b/N10 +V9b

d/dt(S15) = Grow10*S10/N10 - Grow15*S15/N15 -beta15*S15*Itotal/Ntotal + delta15a*V15a/5 + delta15b*V15b/5

d/dt(E15) = beta15*S15*Itotal/Ntotal -alfa*E15 -Grow15*E15/N15 + Grow10*E10/N10

d/dt(I15) = alfa*E15 -sigma*I15 - Grow15*I15/N15 + Grow10*I10/N10

d/dt(R15) = sigma*I15 - Grow15*R15/N15 + Grow10*R10/N10

d/dt(V15a) = -delta15a*V15a/5 -Grow15*V15a/N15 +Grow10*V10a/N10

d/dt(V15b) = -delta15b*V15b/5 -Grow15*V15b/N15 +Grow10*V10b/N10

d/dt(S20) = Grow15*S15/N15 - Death*S20/N20 -beta20*S20*Itotal/Ntotal + delta20a*V20a + delta20b*V20b

d/dt(E20) = beta20*S20*Itotal/Ntotal -alfa*E20 -Death*E20/N20 + Grow15*E15/N15

d/dt(I20) = alfa*E20 -sigma*I20 - Death*I20/N20 + Grow15*I15/N15

d/dt(R20) = sigma*I20 - Death*R20/N20 + Grow15*R15/N15

d/dt(V20a) = -delta20a*V20a -Death*V20a/N20 +Grow15*V15a/N15

d/dt(V20b) = -delta20b*V20b -Death*V20b/N20 +Grow15*V15b/N15

C1=0.35

C2a=0.3

C2b=0.05

VE=0.84

VEa= 0.774789

VEb= 0.9

init S0 = 1075413

init E0 =228.313

init I0 = 94.01123797

init R0 = 2444.292187

N0=S0+E0+I0+R0

Births=1000000-10000*t

sigma = 52

alfa=52*7/17

beta0 = 166.0662

init S1 = 715378.8

init E1= 1898.326

init I1 = 781.6635

init R1 = 25211.84

init V1 = 317658.4

N1=S1+E1+I1+R1+V1

delta = 0.02

beta1 = 1037.84

init S2 =646760.5

init E2= 3745.654

init I2 = 1542.328

init R2 = 85635.62

init V2 = 314169.4

N2=S2+E2+I2+R2+V2

delta = 0.02

beta2 = 1510.041

init S3 = 531591.9

init E3= 5897.037

init I3 = 2428.192

init R3 = 188869.1

init V3 = 310626.6

N3=S3+E3+I3+R3+V3

delta = 0.02

beta3 = 2169.308

init S4 = 379812.9

init E4= 7584.084

init I4 = 3122.858

init R4 = 333196.4

init V4 = 306741.7

N4=S4+E4+I4+R4+V4

delta = 0.02

beta4 = 3470.936

init S5 = 234700.3

init E5 = 7175.577

init I5= 2954.65

init R5 = 491211.6

init V5a = 260534.3

init V5b = 50439.71

N5=S5+E5+I5+R5+V5a+V5b

delta5a =0.1

delta5b = 0.02

beta5 = 5256.027

init S6 = 160043.9

init E6= 5217.452

init I6 = 2148.36247

init R6 = 623889.9406

init V6a = 236732.8

init V6b = 49905.64

N6=S6+E6+I6+R6+V6a+V6b

delta6a =0.1

delta6b = 0.02

beta6 = 5543.541

init S7 = 118306.3

init E7 = 3496.475

init I7 = 1439.725

init R7 = 717180.2

init V7a = 214709.6

init V7b = 49286.32

N7=S7+E7+I7+R7+V7a+V7b

delta7a =0.1

delta7b = 0.02

beta7 = 4971.591

init S8 = 100071.9

init E8 = 2300.558

init I8 = 947.2886

init R8 = 779242.6

init V8a = 194823.5

init V8b = 48696.72

N8=S8+E8+I8+R8+V8a+V8b

delta8a =0.1

delta8b = 0.02

beta8 = 3826.036

init S9 = 95957.06

init E9= 1495.162

init I9 = 615.655

init R9 = 819879.1

init V9a = 176597

init V9b = 48064.6

N9=S9+E9+I9+R9+V9a+V9b

delta9a =0.1

delta9b = 0.1

beta9 = 2565.922

init S10 = 702385.1

init E10 = 2447.679

init I10 = 1007.868

init R10 = 4205635

init V10a = 820901.8

init V10b = 223425.7

N10=S10+E10+I10+R10+V10a+V10b

Grow10=1150000-10000*t

delta10a = 0.1/5

delta10b = 0.1/5

beta10 = 573.867

init S15 = 1211304

init E15= 216.8962

init I15 = 89.31019

init R15 = 4234162

init V15a = 798445.8

init V15b= 217313.8

N15=S15+E15+I15+R15+V15a+V15b

Grow15=1200000-10000*t

delta15a = 0.1/5

delta15b = 0.1/5

beta15 = 29.48701

init S20 = 84938313

init E20 = 719.1628

init I20 = 296.12587

init R20 = 4244183

init V20a = 11549106

init V20b = 3143332

N20=S20+E20+I20+R20+V20a+V20b

Death=1300000+10000*t

delta20a = 0.1/65

delta20b = 0.1/65

beta20 = 1.394298

In case of the one-dose routine program, C1=0.95 throughout the study period. C2a=0.3 and C2b=0.05 to year 4, C2a=0.9 C2b=0.05 from year 4 to year 50.

In case of the one-dose routine program, C1=0.95 throughout the study period. C2a=0.55 and C2b=0.35 to year 4, C2a=0.05 C2b=0.9 from year 4 to year 50.

Appendix B. Age-specific incidence rates in 3 scenarios.
